# The Subjective Value of Product Popularity: A Neural Account of How Product Popularity Influences Choice Using a Social and a Quality Focus

**DOI:** 10.3389/fpsyg.2021.738095

**Published:** 2022-01-19

**Authors:** Robert P. G. Goedegebure, Irene O. J. M. Tijssen, L. Nynke van der Laan, Hans C. M. van Trijp

**Affiliations:** ^1^Marketing and Consumer Behavior Group, Wageningen University, Wageningen, Netherlands; ^2^Division of Human Nutrition, Wageningen University, Wageningen, Netherlands; ^3^Department of Communication and Cognition, Tilburg University, Tilburg, Netherlands

**Keywords:** social influence, popularity and quality, decision neuroscience, fMRI, judgment and decision making, product popularity

## Abstract

Research on social influences often distinguishes between social and quality incentives to ascribe meaning to the value that popularity conveys. This study examines the neural correlates of those incentives through which popularity influences preferences. This research reports an functional magnetic resonance imaging experiment and a behavioral task in which respondents evaluated popular products with three focus perspectives; unspecified focus, focus on social aspects, and focus on quality. The results show that value derived with a social focus reflects inferences of approval and reward value, and positively affects preferences. Value derived with a quality (versus normal) focus reflects inferences of quality and negatively affects preferences. This study provides evidence of two distinct inferential routes on both a neurological level, represented by different regions in the brain, and a behavioral level. These results provide the first evidence that a single popularity cue can in different ways influence the value derived from product popularity.

## Introduction

Popular products are often considered popular for a particular reason. The notion that others found a product worthwhile and bought it is used to evaluate the popular product. People then ascribe to the (popular) product a subjective value derived from the product’s popularity. This value may be expressed either in terms of expectations on how to behave and on getting approval from social peers (Berger and Heath, 2007) or in terms of product functionality (Steinhart et al., 2014). These are the two routes through which popularity aids in value assessment. We refer to these as the social and quality routes, respectively. Researchers often distinguish between these two routes by noting either normative (i.e., social) incentives or informational (i.e., quality) incentives to be derived from choosing a popular option (Deutsch and Gerard, 1955; Cialdini et al., 1990). Thus, at a behavioral level, someone may infer that the popular product has the highest value because it outperforms competitors in terms of social approval (i.e., “others buy it, so it must be the approved choice”) and/or in terms of quality (i.e., “others buy it, so it must be good”).

The subjective value that people perceive and ascribe to products is critical for their behavior toward those products. This is emphasized by a wealth of economic and psychological theories (Kahneman and Tversky, 1979; Sheth et al., 1991) and validated by an increasing body of neuroscientific research on people’s internal valuation system (Levy and Glimcher, 2012; Bartra et al., 2013). For behavioral acts influenced by the behavior of others, activity in brain areas that compute value may play an important role (Falk and Scholz, 2018). For example, activity in these areas has been linked to explanations of how the preferences of others influence one’s own preferences for abstract symbols (Mason et al., 2009), how group choices may determine (un)healthy food choices (Nook and Zaki, 2015), and to predicting actual popularity of songs (Berns and Moore, 2012). Nonetheless, it remains unclear whether the activity in the internal valuation system is the result of an integration of information for the purpose of assessing a product’s normative incentives (i.e., social approval) or its informative incentives (i.e., quality), or both (Toelch and Dolan, 2015). It seems unlikely that in all the above-mentioned studies, the final value derived from popularity is the result of activation in the same regions of the brain. The moment that someone uses popularity to assess a product’s value based on an assessment of either social approval or quality, it is not likely that one single brain region explains the value assessment (Ariely and Berns, 2010). In the current paper, we explore the distinction between neural correlates of normative incentives and informational incentives. Specifically, by using a neuroimaging technique, namely functional magnetic resonance imaging (fMRI), we aim to distinguish between the different neural routes (i.e., social approval vs. quality) through which popularity may determine a product’s value.

The main goal of the current study is to examine the two routes (i.e., normative and informational) of social influence (Deutsch and Gerard, 1955) at a psychological and (converging) neurological level. Methodologically, we extend current neuroscientific research on social influence by presenting product popularity as input in the decision-making process rather than as feedback after the decision. Previous studies on social (normative) influences typically investigate neural activation by providing social feedback after participants have provided responses and then measuring the difference in activation (Falk and Scholz, 2018). In contrast, our current study follows a social input approach. The information that we use to convey popularity in this study is information about the share of participants that expressed interest in products in previous studies. Participants are given information on product popularity at the moment of evaluation to track how popularity guides the value computation and influences behavior. In doing so, we draw upon insights that show how people use a single piece of information for multiple purposes (Hare et al., 2011). This allows us to more clearly distinguish between normative and informational social influence. By using novel insights from neuroimaging, these routes can be studied in an unbiased way, providing a richer understanding of the process of popularity and complementing the use of traditional self-reported scales (Plassmann et al., 2015; Morales et al., 2017). In the current paper, we aim to (1) outline the neural correlates for normative social influences and for informational social influences, (2) assess the extent to which the correlates of these routes may deviate (or overlap), and (3) examine whether the neural routes reflect self-reported inferences of social approval and quality.

The remainder of this paper is structured as follows. In the next section we review literature on popularity and decision making from both (consumer) psychology and neuroscientific literature. The aim of the section is to determine different neural routes through which popularity could aid in the assessment of either a social product value or a functional product value. We present the results of a behavioral task and an fMRI study to uncover these two routes. We conclude the study by discussing our findings and their limitations and several directions for future research.

## Theoretical Framework

### Product Popularity and Social Approval

The normative route of social influence is equated with behaving in accordance with the positive expectations and potential approval of others (Deutsch and Gerard, 1955; Cialdini and Trost, 1998). People may thus look at others to decide which product they should choose so as to make a good impression on others and to connect with their social peers so as to gain approval and avoid disapproval. The desire to connect with others and avoid disapproval can have a strong influence on behavior as evidenced by the classic study of Asch (1956). In his study, Asch demonstrated that despite obvious evidence, participants still go along with the group even though they know it is not the best choice to make. Especially in situations where they share a close connection with others, people tend to follow popular behavior (Berger and Heath, 2008). In general, people want to express themselves and signal who they are and with whom they connect (Escalas and Bettman, 2005). Product popularity may then be a great source of information to determine potential social (dis)approval and to assess a product’s social value.

The concept of social value encompasses different elements related to expectations of others’ approval, such as the perception of the extent to which a product signals an identity or would impress social peers, and whether the choice is deemed appropriate (Sweeney and Soutar, 2001). Neurologically, such social valuation has been equated with activity in the middle medial prefrontal cortex (MMPFC), the dorsal medial prefrontal cortex (DMPFC), precuneus (PC), bilateral temporal junction (TPJ), and in the right superior temporal sulcus (rSTS) (Baek et al., 2017). In particular, activity in the TPJ and STS has been found to reflect thinking about the behavior and opinions of others (Van Overwalle, 2009; Abu-Akel and Shamay-Tsoory, 2011; Schurz et al., 2014). Social evaluation is also concerned with expectations of (how to avoid) social penalties, which suggests that the anterior insula (AI) and anterior cingulate cortex (ACC) are also involved because these reflect the decision to conform to opinions of others while considering social penalties (Berns et al., 2010; Eisenberger, 2012; Cascio et al., 2015). Together, all these named regions of the brain are likely to represent a collective that is involved with decision making that takes into account possible social (dis)approval.

We propose that product popularity evokes inferences of social value. As such, we expect that people who examine a product to assess social approval resulting from a product choice will draw from existing knowledge on what others may think to assess the social consequences of their own choice. In doing so, activation in a neural collective that is equated with thinking about the behavior and opinions of others will influence the preferences that people form. The information that others expressed interest in a product will affect the influence on someone’s preferences. As such, popularity will increase the positive valence of the activation. Furthermore, we propose that the inferences of social value mediate the relationship between popularity and preference and thus expect that the activation in the proposed neural regions plays a mediating role as well. Formally, we hypothesize that:

**H1_**A**_**: Product popularity increases neural activity in the PC, MMPFC, DMPFC, rSTS, AI, TPJ, and the ACC (i.e., neural social approval collective).
**H1_**B**_**: Activity in the neural social approval collective mediates the effect of popularity on behavior.

### Product Popularity and Quality

In their seminal paper on the two forms of social influence, Deutsch and Gerard (1955) define informational social influence as the tendency of people to view information about others’ behavior as evidence for reality. This means that when we see many others behave in a certain way by choosing the same option, we assume that option to be the best one in the choice set. Indeed, people tend to choose what others have chosen because they believe that this gives the best chance of them ending up with the best option as well (Bikhchandani et al., 1998). As evidenced by the literature, this link between product popularity and product quality has been studied extensively and in various ways. For example, Cohen and Golden (1972) demonstrated that coffee is perceived to taste better when one knows others enjoyed it as well. Tucker and Zhang (2011) found that consumers perceive popular wedding dresses to come from the better vendors. Steinhart et al. (2014) demonstrated that popular products are often equated with a higher degree of functionality. These studies suggest that people assume that when others buy a product that that product is a functional, well-made, and qualitatively better product than other products in the choice set.

The information that people draw from popularity on the informational social influence route all relate to elements of perceived functional value. Functional value reflects a product’s quality and encompasses elements such as product workmanship, consistency of quality, and quality reliability (Sweeney and Soutar, 2001). One way to arrive at such an evaluation of quality is to evaluate the product on attributes one deems critical for proper functioning. For example, if one is considering buying a chair, one will likely want to know how it sits. Similarly, if one is contemplating buying a box of cookies, one will likely think about how those cookies will taste. In order to form those expectations, one needs to rely on the knowledge they have about that particular product option.

In neurological terms, knowledge about objects (i.e., products) is stored in the lateral and ventral regions of the temporal cortex (Chao et al., 1999). These regions serve as a personal library that stores information about the objects that people know and the elements they find critically relevant for the performance of an object. For that object to be deemed “better in performance,” people will look for positive features in the product. In consumer settings, the temporal cortex also aids people to assess the functional value of products by considering the different product attributes (Couwenberg et al., 2017). The integration of this information about the attributes is often reflected in activity in the (medial) orbitofrontal cortex and the medial prefrontal cortex (mPFC) (Knutson et al., 2007; Plassmann et al., 2008; Rushworth and Behrens, 2008; Karmarkar and Yoon, 2016). In particular, the medial OFC (mOFC) is known to be active when it comes to assessing and integrating information on the functionality of a choice for calculating a subjective reward value (Kringelbach, 2005; Hare et al., 2008). Moreover, the mOFC has been specifically implicated as part of the assessment of product quality (Rangel et al., 2008). Thus, people who use popularity to assess product quality are likely to display activity in a collective of brain regions that is comprised of a combination of the temporal cortex (ventral and lateral regions), the mOFC, and the mPFC.

Product popularity is proposed to evoke inferences of quality. We expect that people who examine a product to assess product quality will draw on existing knowledge about the specific properties of a product to derive an outcome value. In doing so, what informs someone’s preferences is the activation in a group of brain regions that is equated with functional benefits and the integration of information on marketplace offerings. Information that other consumers have expressed interest in those marketplace offerings will affect the informing of individual preferences. As such, popularity will increase the positive valence of the activation. Furthermore, we propose that the inferences of quality mediate the relationship between popularity and preference and thus expect that the activation in the proposed neural regions plays a mediating role as well. Formally, we hypothesize:

**H2_*A*_**: Product popularity increases activity in the temporal cortex, the OFC, and the mPFC (i.e., neural quality collective).
**H2_*B*_**: Activity in the neural quality collective mediates the effect of popularity on behavior.

### Reward Value of Product Popularity

The popular option may be considered the better choice in terms of quality and/or social approval. Regardless of the type of inference people draw from the product’s popularity, the final result will likely be an assessment of the product’s total subjective (i.e., reward) value. Prior studies have produced neurological evidence that implicates specific regions in the brain to make up an internal valuation system (Levy and Glimcher, 2012; Bartra et al., 2013). This system consists of parts of the striatum (both left and right), the ventromedial prefrontal cortex (vmPFC), portions of the ACC and PCC, and parts of the anterior insula (both left and right). Activities in these regions have been linked to expecting and receiving different types of reward. For example, the vmPFC has been found to encode the value for money, snacks, and different trinkets such as memorabilia (Chib et al., 2009). More importantly, activity in regions of the valuation system has also been linked with socially influenced behavior (Bhanji and Delgado, 2014). People who learn that others agree with their evaluations tend to display greater activity in portions of the striatum (Klucharev et al., 2009; Zaki et al., 2011). Thus, the popular option may evoke activity in the valuation system. Nevertheless, we expect that this activity will be accompanied by activity in the neural quality collective and/or the neural social approval collective, depending on the inferences that people draw from popularity to inform their decisions.

The inferences that inform people’s decisions may be situationally specific when they make a decision in particular situations with a mindset focused on a certain outcome. Individual preferences and choices are influenced by the mindset they hold because the mindset determines how available information is used (Xu and Wyer, 2007; Wyer, 2008). Subsequent behavior is then informed by someone’s focus on the information in their environment at that moment (Kahneman and Miller, 1986; Loersch and Payne, 2011). In the case of product popularity, this means that people who are focused on social value will more likely activate the use of the normative aspects of popularity to infer social approval, whereas a focus on product quality will more likely use the informational aspects of popularity to infer quality. On a neurological level, we expect that this is reflected by distinctive brain activation in the neural quality collective and the neural social approval collective. On a behavioral level, the routes have been proposed to share properties (Cialdini and Goldstein, 2004). Yet, we propose that the two brain collectives, that reflect the quality and social routes, each have a distinctive contribution to the value computation.

**FIGURE 1:**
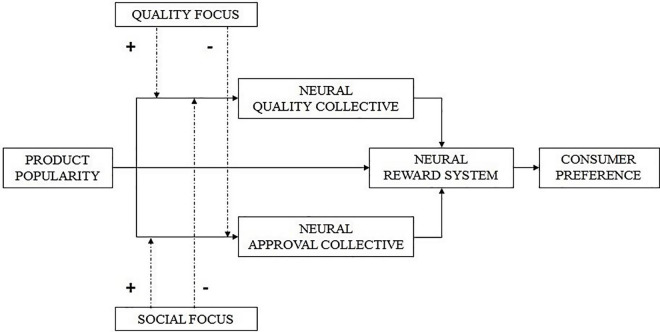
Conceptual framework. The effect of product popularity on consumer preference is mediated by inferences of quality, reflected by activity in the neural quality collective, inferences of social approval, reflected by activity in the neural approval collective, and activity in the neural reward system. The magnitude of the mediating effect is dependent upon the focus condition.

For the subjective value of popularity, this means that when one focuses on the social aspects of the situation, the combination of encoding value in both the social and reward systems is expected to occur; whereas the combination of encoding value in both the quality and reward systems is expected to occur when one focuses on quality. In psychological terms, we propose that for people with a social focus, the effect of popularity on preference is mediated by inferences of social value, whereas for people with a quality focus, the effect of popularity on preference is mediated by inferences of quality. We expect that this pattern holds on a neurological level. Formally, we hypothesize the following (please see Figure 1 for a full overview of the conceptual framework):

**H3**: For people with a social focus, the route from popularity to behavior is expressed through heightened activity in both the social approval neural collective and the reward system; whereas for people with a quality focus, the route from popularity to behavior is expressed through heightened activity in both the neural quality collective and the reward system.

## Materials and Methods

### Method

Thirty-three participants took part in the study. Two participants were excluded due to a task programming error. One participant was excluded because she had a panic attack during data collection. We present data from 30 young (*M*_*age*_ = 22.2), right-handed female participants. We included female participants only because female brains may respond differently to food stimuli than male brains (Frank et al., 2010). To avoid potential interferences with the collection of brain images, participants were screened on several aspects. First, none of the participants reported a history of drug abuse, head trauma, neurological, or psychiatric illness. Second, they were screened to ensure that they were not currently following any specific diet or seeking to lose weight for any reason, or taking medications that could interfere with the performance of fMRI.

Participants were told during the first information meeting that they would be part of a large pool of participants who evaluated products in earlier versions of the current study. Unbeknownst to the participants, this was a fictitious subject pool and part of our manipulations (including the manipulation of popularity). The demographics of the subject pool were: female, Dutch native speakers, age 18–35 years old, student, healthy BMI; their lifestyle-related characteristics were: regular supermarket visits, doing your own cooking and grocery shopping, variation in dietary habits, strong opinion, and caring and social. The description of the subject pool was pretested, as described in the Supplementary Data File.

A critical pre-requirement for inclusion in the current study was for a participant to have a strong association with the social group that comprised the fictitious subject pool. This was to increase the effectiveness of our popularity manipulation. Group association was measured with three (1–9 point scale) statements (adapted from Escalas and Bettman, 2005) during the information meeting. Mean scores (> 7) were used as decision criteria to invite participants for further participation.

#### Design

**FIGURE 2:**
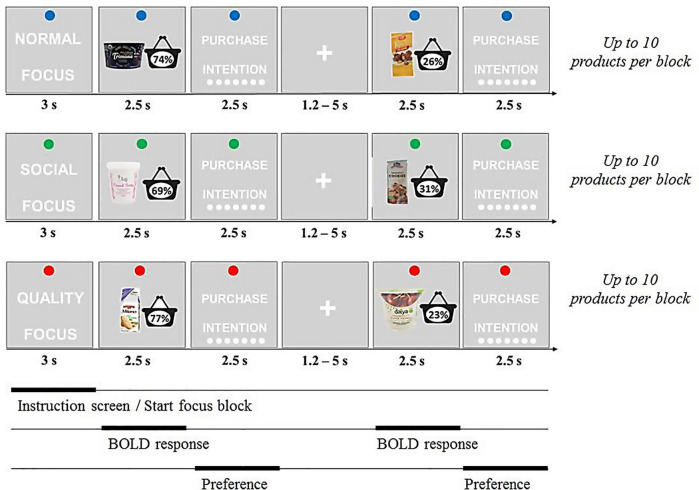
Overview of experimental design. Participants expressed purchase intention in three conditions: (1) normal focus condition, (2) social focus condition, and (3) quality focus condition. Before each block of 10 products, subjects were informed which aspect of the product to pay attention to before making the choice.

The experiment followed a 3 (focus) × 2 (popularity) within-subjects design (see Figure 2 for an illustration). Participants were asked to express their purchase intention for 180 food products, divided over three different focus conditions (cf. Hare et al., 2011). The task was incentive-based, meaning that participants were told that at the end of the study they would get one of the products that rated high in their purchase intention scores. To manipulate the focus condition and induce situation-specific mindsets, we asked participants to read different scenarios that represented the focus conditions (please see Supplementary Data File for full details). In the normal focus condition, the participants were asked to evaluate the products in the way they would usually do. In the social focus condition, the participants were asked to focus on the potential social value of the products and whether or not purchasing them would help to impress others and gain approval from social peers. In the quality focus condition, the participants were asked to focus on the products’ quality-related properties and to assess whether or not the products were of high quality. In order to warrant a proper understanding of the manipulations, participants were instructed and informed about the conditions at the start of the training session, before the scan session and again before the post-scan session.

The products presented in each condition where either low or high in popularity (pretest details are reported in the Supplementary Data File). Product popularity was manipulated by presenting to the participants the “popular choice scores” of the products, which were derived from previous studies (i.e., the fictitious subject pool). For each product, participants were shown a shopping basket reflecting the percentage of previous participants who wanted to buy that product. Low percentages represented low popularity, high percentages represented high popularity. To increase credibility and avoid repetition of percentages, percentages differed within a range (low: 23–34%; high: 66–77%). Percentages were embedded in the product image, but combinations with popularity (low/high) were counterbalanced.

Participants evaluated a total of 180 products that were equally distributed across the six conditions. To avoid order and timing effects that could interfere with brain activity, participants evaluated the products in 18 blocks of 10 products (five high in popularity, five low in popularity) within a particular focus. Each of these focus blocks was introduced with an instruction screen (3 s) to announce the focus of that condition. For example, participants first evaluated 10 products with a quality focus, and next they evaluated 10 products with a normal focus. The order in which these blocks were presented was randomized and counterbalanced across participants. The order of product presentation was fully randomized. Thus, each participant evaluated a set of 180 products in a unique order.

#### Stimuli

One hundred eighty digital photos of supermarket products were collected for the study. Stimuli were collected from an online database and selected based on the criteria “introduction in the market from 2011 to 2015” and commercial unavailability in the local market at the time of data collection. Products were selected from the categories: cookies, desserts, sodas, and cheeses. These categories were chosen because they could be part of regular grocery shopping and participants likely had experience with these types of products.

#### Procedure

Each participant took part in two sessions on two separate days, with 2–14 days in between. The first session entailed a training session at the university campus. The second session involved the scan session at the hospital where the university’s scanner is located. In the first session, the participants completed the following scales: trait conformity (Mehrabian and Stefl, 1995), susceptibility to interpersonal influence (Bearden et al., 1989), need to belong (Baumeister and Leary, 1995), association with the subject pool (Escalas and Bettman, 2005), and consumer need for uniqueness (Tian et al., 2001; Ruvio et al., 2008). These measures were not used in the present study but were part of a different study design that tested the effects of these personality traits on the processing of social information. Next, the participants practiced the scan task in a mock Magnetic Resonance Imaging (MRI) scanner to get them accustomed to participating in an fMRI experiment. The stimuli used in this first session were different from those used in the main study. As the first session was purely instructional for the main study, we did not collect any imaging data during this session. No participants dropped out after this session.

During the main scan session, the participants evaluated 180 products in consecutive trials in an MRI scanner (see Supplementary Data File for magnetic resonance data acquisition parameters). Each trial started with the presentation of a picture of a product for 2.5 s. Next, participants indicated their purchase intention using a 7-point scale (1–7; starting point randomized) via a button box (2.5 s). Participants were then shown a fixation cross of jittered length (1.2–4.8 s). This completed the product trial. After completing 180 product trials, the participants continued with a different task (unrelated to the current study) in the scanner. Stimuli were presented and responses were collected with use of the Presentation^®^ software (Neurobehavioral Systems Inc., Albany, CA, United States).

To validate the results obtained in the scanner, we asked the participants to complete an adapted and shortened version of the scan task after they had left the scanner. In this task, they expressed their purchase intention for a subset of the products from the scan task and evaluated each product in the subset in terms of social approval (cf. Sweeney and Soutar, 2001), quality (cf. Sweeney and Soutar, 2001), and popularity (“This product is popular”). Inferences were measured by asking participants to indicate their agreement to statements that reflected inferences (e.g., “This product is of good quality”). Responses were collected with 9-point scales (disagree-agree). This provided the additional behavioral measures to link inferences of quality and social value to neural correlates. We opted for a shortened version of the scan task because the full version would have been too strenuous for the participants. In each of the 18 blocks, the 2nd and 4th popular products and the 2nd and 4th unpopular ones were selected from a participants’ product set. This comprised a total of 72 evaluations; a subset of the 180 products that participants evaluated in the scanner. Participants completed the task at their own pace (i.e., no time restraints) using a computer on which the products were presented visually identical to how they were presented in the scanner. At the end of this task, the participants completed the Consumers’ Need For Uniqueness scale (Tian et al., 2001) and the Consumer Susceptibility to Interpersonal Influence scale (Bearden et al., 1989).

### Magnetic Resonance Imaging Data Analysis

#### Subject-Level Analysis

Neuroimaging data were first preprocessed, as detailed in the Supplementary Data File. The next preparatory step for analysis included, per participant, a specification of the design and the parts of each trial. Six conditions were modeled (c.f., 3 focus × 2 popularity study design) for the moments of product evaluation (i.e., stimulus presentation): normal focus + popular products; normal focus + unpopular products; social focus + popular products; social focus + unpopular products; quality focus + popular products; and quality focus + unpopular products. Participants also saw visuals such as the instruction screen (3 s) and the response screen for purchase intention (2.5 s). We were not interested in the activity during these moments, so these moments were modeled as regressors of no interest. Finally, realignment parameters were added to account for variance that resulted from head movements in the scanner.

We estimated two models. The first model (i.e., model 1) was estimated exactly as described above. In the second model (i.e., model 2), purchase intention was included as a parametric modulator of the moments of evaluation. This second model examined the brain activity at the moment of evaluation that was correlated with the purchase intention in the six different study conditions. One participant was dropped from the data for this model because of a lack of variance in responding in one of the conditions, leaving a sample of 29 participants for model 2.

#### Regions of Interest

To test our hypotheses, we created three masks with regions of interest (ROI) that reflected the brain regions from our hypothesized collectives. One mask was created per construct (quality, social approval, reward). All masks were created based *a priori* on the coordinates of peak activation found in previous studies, as suggested by several position papers (Poldrack, 2007; Smeets et al., 2019). For the reward value ROI mask, peak coordinates were taken from a meta-analysis by Bartra et al. (2013). The quality ROI mask was created using peak coordinates from Hare et al. (2008) and Couwenberg et al. (2017). The social approval ROI mask was built from peak coordinates taken from Berns et al. (2010) and Baek et al. (2017). Peak coordinates were used to create 10mm spheres using the WFU PickAtlas tool in SPM 12 (Maldjian et al., 2003). Each study was selected because of its close fit, methodologically and theoretically, with the current research. Please see the Supplementary Data File for a full description per study, including an overview of all the peak coordinates that were used.

**FIGURE 3:**
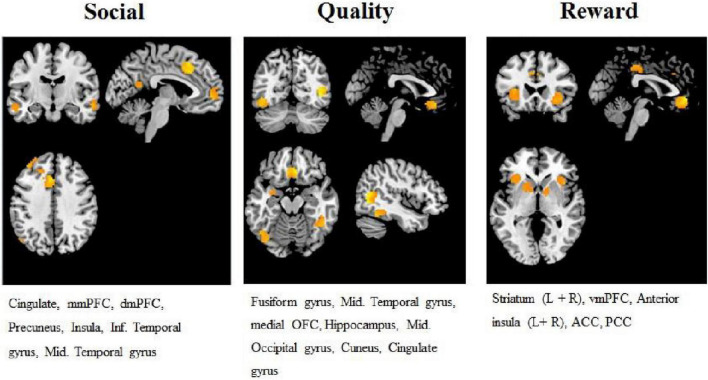
Overview of regions of interest (ROIs) masks used in the analyses. The regions that are included per mask are listed below the mask. The specific ROIs per mask are the yellow/orange areas.

Next, to exclude inactive voxels from our ROI, we thresholded the masks on the overall treatment effect (i.e., activation at the moments of evaluation in all six conditions versus baseline). We opted for a more stringent threshold and chose FWE correction with *p* < 0.05, with *k* > 42 (calculated with SPM ClusterSize Threshold) to ensure that the significant voxels were meaningful and to decrease the probability of false positives. The resultant masks (please see Figure 3 for an overview) contained roughly the same regions as hypothesized, which indicated activity in all regions throughout the experiment. The social ROI was comprised out of regions within the mmPFC, dmPFC, PC, ACC, AI, and the inferior and mid temporal gyrus. The quality ROI was comprised out of regions in the middle temporal gyrus, mOFC, cuneus, middle occipital gyrus, cingulate gyrus and the hippocampus. Finally, the reward ROI consisted out areas of the striatum (both left and right), vmPFC, ACC and PCC, and parts of the anterior insula (both left and right).

#### Data Extraction

The masks were used to subtract the percentage signal change between conditions for several prespecified contrasts to create parameters for testing the mediation hypotheses. Parameters were extracted from contrasts in which activity in the focus conditions (social and quality) was corrected for the activity in the normal focus conditions (model 1 without purchase intention as parametric modulator). Parameters were extracted from the following conditions: “social focus and popularity high” versus “normal focus and popularity high;” “social focus and popularity low” versus “normal focus and popularity low;” “quality focus and popularity high” versus “normal focus and popularity high;” “quality focus and popularity low” versus “normal focus and popularity low.” Extraction was done using the MarsBar toolbox for SPM (Brett et al., 2002). Whole brain analyses are reported in the Supplementary Data File.

## Results

### Behavioral Results

We examined the effect of popularity on behavior mediated by inferences of quality and social approval, with the propensity of the inferences moderated by the focus conditions via a multilevel model using a moderated mediation approach (Muller et al., 2005). The model was estimated with purchase intention (*Y*_*Intent*_) as dependent variable. The model included popularity (*X*_*Pop*_) as main independent variable, social focus (*MO*_*So*_) and quality focus (*MO*_*Qual*_) were included as moderators, inferences of social approval (*ME*_*Soc*_) and inferences of quality (*ME*_*Qual*_) were included as mediators. Due to the hierarchical nature of the model and to avoid misinterpretation of the parameters, the categorical predictors that indicated the quality focus and social focus were effect-coded (O’Grady and Medoff, 1988; Bech and Gyrd-Hansen, 2005), where focus in both cases was coded as 1, and normal focus as –1. All interactions necessary for testing for moderated mediation were included. The analyses were conducted with SPSS (Version 25, IBM Corp., Armonk, NY, United States) on the data that were collected in the post-scan task in which participants evaluated a subset of the products from the scan task.

The effect of popularity on purchase intention and the mediators was first tested with separate models. These results (see Table 1 for full overview) largely comply with the requirements for testing moderated mediation (Muller et al., 2005). The results for the complete model show that both inferences of social approval (β*ME*_*Soc*_ = 0.33, *p* < 0.001) and quality (β*ME*_*Qual*_ = 0.49, *p* < 0.001) mediated the relationship between popularity and purchase intention. The interaction between the social focus and inferences of social approval was significant and in the expected direction (β*ME_*Soc*_* × *MO_*Soc*_* = 0.11, *p* = 0.011). This indicates that social inferences are particularly influential on purchase intention when people hold a social focus. There was no significant interaction between the quality focus and inferences of quality (β*ME_*Qual*_* × *MO_*Qual*_* = 0.01, *p* = 0.749). Nonetheless, the results showed a significant negative interaction between the social focus and inferences of quality (β*ME_*Qual*_* × *MO_*Soc*_* = –0.10, *p* = 0.029). This indicates that the effectiveness of the inferences of quality varies as a function of whether participants hold a social versus normal focus. More specifically, these findings indicate that participants easily inferred product quality from popularity in both the normal and quality focus and that the inferences of quality influenced choice, but that these influential effects were attenuated within a social focus. Finally, the overall treatment effect of popularity on purchase intention remained significant but became negative (β*X*_*Pop*_ = –0.23, *p* < 0.001), which indicated partial mediation.

**TABLE 1:** Overview test statistics and parameters for behavioral focus effects.

Model	DV: Y_Intent_		DV: ME_Qual_		DV: ME_Soc_		DV: Y_Intent_	
*Predictors*	β*(SE)*	*t*	β*(SE)*	*t*	β*(SE)*	*t*	β*(SE)*	*t*
Intercept	4.99 (0.17)	30.18***	5.44 (0.11)	47.46***	5.17 (0.11)	45.96***	0.60(0.18)	3.37**
X_Pop_	0.37 (0.05)	7.45***	0.61 (0.04)	15.34***	0.92 (0.04)	21.76***	–0.23(0.04)	–5.37***
MO_Soc_	–0.02 (0.07)	–0.33	–0.00 (0.06)	–0.05	0.13 (0.06)	2.17*	–0.09(0.16)	–0.56
X_Pop_*MO_Soc_	–0.06 (0.07)	–0.79	–0.12 (0.06)	–2.09*	–0.18 (0.06)	–2.97**	0.02(0.06)	0.40
MO_Qual_	–0.11 (0.07)	–1.53	–0.10 (0.06)	–1.79^†^	–0.12 (0.06)	–2.00*	–0.14(0.16)	–0.87
X_Pop_*MO_Qual_	0.05 (0.07)	0.66	0.10 (0.06)	1.70^†^	0.06 (0.06)	1.05	–0.05(0.06)	–0.79
ME_Soc_							0.33(0.03)	10.99***
ME_Socl_*MO_Soc_							0.11(0.04)	2.56**
ME_Soc_*MO_Qual_							0.01(0.04)	0.26
ME_Qual_							0.49(0.03)	15.35***
ME_Qual_*MO_Soc_							–0.10(0.05)	–2.18*
ME_Qual_*MO_Qual_							0.01(0.04)	0.32
Intercept [PP]^a^	0.75 (0.21)	3.53***	0.35 (0.10)	3.41**	0.33 (0.10)	3.33**	0.49(0.14)	3.55***

*^a^Wald Z instead of t.*

***p < 0.001; **p < 0.01; *p < 0.05; ^†^p < 0.10.

Overall, these results indicate a fairly prototypical case of moderated mediation (Muller et al., 2005). This is because the results depict an unmoderated overall treatment effect (i.e., no effect of the focus on the main effect of popularity), yet do show moderated indirect effects via the mediators on the outcome variables. These results provided partial behavioral validation for the hypotheses of this paper: there were the expected effects of social approval in the social route and an attenuation of this effect in the quality route. However, the behavioral results did not show the expected increased effects of inferences of quality when people hold a focus on quality.

### MRI Social Focus

To examine the neural correlates of popularity in the social focus condition, we examined the activation in the *a priori* defined ROI in the social focus condition, while correcting for activation in the normal focus condition. Parameters were extracted (as described above) and entered in a multilevel mediation model of the scores of purchase intention that were collected in the MRI scanner. Popularity (*X*_*Pop*_) was entered as an independent variable, with the following mediators: parameters of activity in the social approval collective (*ME*_*ROI_Soc*_), parameters of activity in the quality collective (*ME*_*ROI_Qual*_), parameters of activity in the reward system (*ME*_*ROI_Rew*_), and interactions between the parameters of social approval and reward (*ME_*ROI_Soc*_* × *ME_*ROI_Rew*_*) and quality and reward (*ME_*Qual*_* × *ME_*Rew*_*). Because we corrected for brain activity in the normal focus condition, there was no need for moderator variables. Different parts of this multilevel model were analyzed in a stepwise fashion to examine mediation before we ran the full multilevel model (see Table 2 for a full overview of results). The mediation analyses were conducted with SPSS (Version 25, IBM Corp., Armonk, NY, United States).

**TABLE 2:** Parameters and test statistics for social focus.

Mediators only; *Y* = Intent					
***Fixed parameters***	β	***SE***	***df***	***t***	***p***

Intercept	–0.100	0.082	30.015	–1.209	0.236
X_Pop_	0.750	0.037	1793.817	20.154	0.000
ME_Qual_ROI_	0.084	0.191	230.061	0.440	0.661
ME_Soc_ROI_	0.372	0.156	90.370	2.390	0.019
ME_Rew_ROI_	–0.534	0.180	129.562	–2.968	0.004

***Covariance parameters***	**β**	***SE***	***Wald Z***		***p***

Error term – model	2.380	0.080	29.732		0.000
Error term – intercept	0.153	0.051	2.972		0.003

**Mediators only; *Y* = Reward ROI**					

***Fixed parameters***	**β**	***SE***	***df***	***t***	***p***

Intercept	0.042	0.060	29.959	0.697	0.491
X_Pop_	0.022	0.004	1773.187	6.106	0.000
ME_Qual_ROI_	–0.210	0.022	1791.234	–9.628	0.000
ME_Soc_ROI_	0.698	0.015	1796.116	48.128	0.000

***Covariance parameters***	**β**	***SE***	***Wald Z***		***p***

Error term – model	0.021	0.001	29.748		0.000
Error term – intercept	0.107	0.028	3.841		0.000

**Full model incl. interactions**					

***Fixed parameters***	**β**	***SE***	***df***	***t***	***p***

intercept	–0.242	0.103	30.671	–2.349	0.025
X_Pop_	0.764	0.037	1783.201	20.506	0.000
ME_Qual_ROI_	0.062	0.200	314.433	0.312	0.756
ME_Soc_ROI_	0.368	0.172	99.565	2.138	0.035
ME_Rew_ROI_	–0.479	0.194	153.182	–2.466	0.015
ME_Qual_ROI_ × ME_Rew_ROI_	–0.387	0.442	506.447	–0.877	0.381
ME_Soc_ROI_ × ME_Rew_ROI_	0.875	0.224	807.684	3.907	0.000

***Covariance parameters***	**β**	***SE***	***Wald Z***		***p***

Error term – model	2.347	0.079	29.678		0.000
Error term – intercept	0.230	0.077	2.980		0.003

We first examined the main effects of the different ROI on purchase intention. We found that activity in the social approval collective positively contributed to purchase intention (β*ME*_*ROI_Soc*_ = 0.372, *p* = 0.019). Activity in the quality collective did not contribute to purchase intention (β*ME_*ROI_Qual*_* = 0.084, *p* = 661). Activity in the reward system contributed negatively to purchase intention, meaning that more activity in the reward system signaled a lower purchase intention (β*ME_*ROI_Rew*_* = –0.534, *p* = 0.004). The main effect of popularity remained a positive contributor to purchase intention (β*X_*Pop*_* = 0.750, *p* < 0.001). Next, the relation between activity in the regions of social approval and reward value and activity in the regions of quality and reward value were analyzed. The results of a multilevel model showed that activity in the social approval collective positively affected activity in the reward system (β*ME*_*ROI_Soc*_ = 0.698, *p* < 0.001). Nevertheless, activity in the quality collective negatively affected activity in the reward value system (β*ME_*ROI_Qual*_* = –0.022, *p* < 0.001). Albeit small, popularity positively affected activity in the reward value system (β*X_*Pop*_* = 0.022, *p* < 0.001). For the final model that included all mediators and interactions, the results showed that activity in the social approval collective positively contributed to purchase intention (β*ME*_*ROI_Soc*_ = 0.368, *p* = 0.035). Moreover, there was a significant interaction between the parameters of social approval and reward value, which positively linked to purchase intention (β*ME_*ROI_Soc*_* × *ME_*ROI_Rew*_* = 0.875, *p* < 0.001). These results were in line with our expectations. However, contrary to our expectations, the model also showed a negative effect of activity in the reward system on purchase intention (β*ME_*ROI_Rew*_* = –0.479, *p* = 0.015). The main effect of popularity remained significant (β*X_*Pop*_* = 0.764, *p* < 0.001). None of the other predictors reached significance (*p*s > 0.10).

Thus, in the social focus condition, the effect of popularity on purchase intention may be explained by activity in the social approval collective and by an interaction between activity in this collective and in the reward value system. The parameters obtained here contributed positively to explaining purchase intention. Contrary to our expectations, activity in the reward value system by itself did not positively affect purchase intention. Reward value appeared only to be a positive predictor when accompanied by activity in social value regions.

### MRI Quality Focus

The neural correlates of popularity in the quality condition were examined using a similar approach as in the social focus condition. Here, we examined the activation in our set of *a priori* defined ROI in the quality focus condition and compared this to activation in the normal focus condition (see Table 3 for a full overview of results). The results of the direct effects revealed that activity in the quality regions negatively affected purchase intention (*ME*_*ROI_Qual*_ = –0.438, *p* = 0.052), and so did activity in the reward system (β*ME*_*ROI_Rew*_ = –0.945, *p* < 0.001). Activity in the social regions positively contributed to purchase intention (β*ME*_*ROI_Soc*_ = 0.533, *p* = 0.001). Popularity also positively affected purchase intention (β*X*_*Pop*_ = 0.718, *p* < 0.001).

**TABLE 3:** Parameters and test statistics for quality focus.

Mediators only; *Y* = Intent					
***Fixed Parameters***	**β**	***SE***	***df***	***t***	***p***

Intercept	–0.108	0.094	32.100	–1.156	0.256
X_Pop_	0.718	0.038	1729.087	19.099	0.000
ME_Qual_ROI_	–0.438	0.224	158.758	–1.957	0.052
ME_Soc_ROI_	0.533	0.161	152.330	3.313	0.001
ME_Rew_ROI_	–0.945	0.221	174.210	–4.282	0.000

***Covariance parameters***	**β**	***SE***	***Wald Z***		***p***

Error term – model	2.248	0.076	29.728		0.000
Error term – intercept	0.199	0.064	3.133		0.002

**Mediators only; Y = Reward ROI**					

***Fixed parameters***	**β**	***SE***	***df***	***t***	***p***

intercept	–0.019	0.048	30.156	–0.389	0.700
X_Pop_	0.024	0.003	1775.538	7.549	0.000
ME_Qual_ROI_	0.322	0.023	1799.894	14.107	0.000
ME_Soc_ROI_	0.553	0.012	1799.680	47.929	0.000

***Covariance parameters***	**β**	***SE***	***Wald Z***		***p***

Error term – model	0.015	0.001	29.748		0.000
Error term – intercept	0.068	0.018	3.854		0.000

**Full model incl. interactions**					

***Fixed parameters***	**β**	***SE***	***df***	***t***	***p***

intercept	–0.144	0.102	31.530	–1.405	0.170
X_Pop_	0.703	0.039	1696.383	17.975	0.000
ME_Qual_ROI_	–0.515	0.239	152.713	–2.157	0.033
ME_Soc_ROI_	0.557	0.167	162.327	3.333	0.001
ME_Rew_ROI_	–0.798	0.241	222.676	–3.320	0.001
ME_Qual_ROI_ × ME_Rew_ROI_	–0.098	0.334	852.955	–0.292	0.770
ME_Soc_ROI_ × ME_Rew_ROI_	0.220	0.160	1126.427	1.372	0.170

***Covariance parameters***	**β**	***SE***	***Wald Z***		***p***

Error term – model	2.246	0.076	29.689		0.000
Error term – intercept	0.238	0.077	3.102		0.002

The results of the activity in the reward system showed that all factors positively contributed to activity in the reward system. Thus, popularity positively linked to activity in the reward system (β*X*_*Pop*_ = 0.024, *p* < 0.001), activity in the quality collective evoked more activity in the reward system (*ME*_*ROI_Qual*_ = 0.322, *p* < 0.001), and activity in the social approval collective evoked more activity in the reward system (β*ME*_*ROI_Soc*_ = 0.553, *p* < 0.001). The full model, which included all effects and interactions, deviated from our expectations. We found that purchase intention was negatively affected by activity in the quality collective (*ME*_*ROI_Qual*_ = –0.515, *p* = 0.033). Purchase intention was also negatively affected by activity in the reward system (β*ME*_*ROI_Rew*_ = –0.798, *p* = 0.001). This was contrary to our expectations. Purchase intention was positively affected by activity in the social approval collective (β*ME*_*ROI_Soc*_ = 0.557, *p* = 0.001). The main effect of popularity remained significant (β*X*_*Pop*_ = 0.703, *p* < 0.001). Neither of the interaction effects reached significance (*p*s > 0.10).

Thus, in the quality focus condition the positive effect of popularity on purchase intention was not explained by either activity in the quality collective or by activity in the reward system. This was contrary to what we expected: we expected that in the quality focus condition activities in both the quality collective and the reward system and an interaction between these two would positively contribute to purchase intention. However, only the activity in the social approval collective positively affected purchase intention. These findings will be further discussed in the general discussion section below.

### Assessing Potential Overlap

The current study distinguishes between a collective for approval and popularity and a neural collective for quality and popularity. At a consumer behavior level, it has been argued that the inferences that reflect the proposed activation share similar properties and may prove to be hard to separate on a neural level (Schnuerch and Gibbons, 2014). The results discussed above demonstrate that the two routes have distinctive effects on preference; both on a behavioral and on a neural level. To test whether this holds at the neural level and assess the extent to which the correlates may deviate (or overlap), conjunction analyses were applied to test for overlap in the regions that are correlated with purchase intention (Nichols et al., 2005). The conjunction analyses (*p_*uncorrected*_* < 0.005; *k* > 20) combined the following contrasts: “quality focus and low popularity versus baseline” and “social focus and low popularity versus baseline” and the contrasts, “quality focus and high popularity versus baseline” and “social focus and high popularity versus baseline.” The contrasts used for the conjunction analyses were all from the model with purchase intention as parametric modulator (model 2), in order to control for correlations with purchase intention. For the combination of the high popular conditions, the conjunction analysis identified a part of the lingual gyrus that was active in both the social and quality focus conditions when evaluating products high in popularity ([*x* = 9, *y* = –76, *z* = −4], *k* = 104, *p_*FWE*_* < 0.05). The combination of the low popular conditions (social focus and quality focus) did not produce significant activation. The results indicate that the neural routes proposed for quality and approval only shared some activation for the evaluation of products high in popularity. However, the lingual gyrus is often linked to tasks such as visual and word processing, and memory activity (Leshikar et al., 2012). It is thus uncertain whether the activity in that region can be attributed to product popularity.

## General Discussion

Decision making is often heavily influenced by the choices and preferences of others, that is, by product popularity. The current study demonstrates the existence of two forms of social influence at a biological level and offers neuroscientific insights into the subjective value that people derive from product popularity. The study demonstrates that a single piece of information may be processed through different neural routes that reflect inferences of quality or of social approval. The results of the route of social approval on a neural level are parallel to the results on the behavioral level. We found that participants who hold a social focus, compared to those who hold a normal focus, use popularity to assess expectations of social approval of the product choice, and that these expectations positively affect their purchase intention. Furthermore, we found that activity in this social approval collective positively interacted with activity in the reward system and subsequently had a positive effect on the participants’ preferences. These results are in line with our expectations. The current study also produced results opposite to what we had expected. We expected that with a quality focus, compared to a normal focus, activity in the quality collective would drive the effect of popularity on choice. The results show the opposite: the activity in the quality collective negatively affected purchase intention. This pattern of the neural results is partially reflected by the pattern of the inferences that were measured in the behavioral portion of the study. These latter results confirm that, similar to the neural results, people’s preferences are indeed more informed by inferences of social approval when they hold a social focus. Moreover, the effect of inferences of quality was attenuated in this (social) focus. The inferences of quality did mediate the effect of popularity on preference, but this effect was not boosted when people held a quality focus. This suggests that inferences of quality are made upon noting popularity in a normal focus condition, but not in a quality focus condition. Finally, the results of two conjunction analyses confirm that one can distinguish two separable neural routes of social influence.

On both the quality and social route the results show that activity in the reward system negatively contributes to purchase intention. This finding is in contrast with previous literature that shows that activity in the reward system positively influences purchase intention (e.g., Knutson et al., 2007). In a follow-up analysis we explored the negative interactions (please see the Supplementary Data File for full analyses and statistics). First we examined activity in the normal focus conditions corrected for activity in the social focus conditions. Those results show that the effect of popularity on purchase intention was in part explained by activity in the quality collective, as well as an interaction between the quality collective and the reward system. The interaction between the social approval collective and the reward system negatively affected purchase intention. These results support our previous argument that the subjective value of popularity in itself may easily be equated with quality and functional benefits. Next, we examined activity in the normal focus conditions while correcting for activity in the quality focus conditions. Here, the effect of popularity on purchase intention was for a large part explained by activity in the reward system. This is in line with previous research that positively links activity in the reward system to purchase intention. Finally, activity in the social approval collective interacted with activity in the reward system and negatively affected purchase intention. It appears that when in a normal focus situation, popularity may not be a product attribute from which people infer social approval. Together, the follow-up analyses suggest that the negative effect of the reward system that we report above, is the result of correcting for the activity during normal focus. The correction of activity in the normal focus condition was executed to rule out extraneous effects or unrelated confounds to measure activity in brain regions that are specifically involved with the two routes of social influence. A disadvantage appears to be that we uncovered a negative relation between activity in the reward system and purchase intention. However, this does not interfere with the aim of our study; to uncover the neural correlates specific to the quality route and the social approval route.

The findings of this research add to the understanding of social influences in several ways. First, prior neuroscientific research on social influences often examined the impact of a popularity cue after the initial evaluation. By examining the initial response to popularity cues, we demonstrate the initial inferences that the cue evokes and offer insights beyond the conformity perspective. People do not only conform to the behavior of others after receiving feedback; they also tend to favor the popular option at the initial moment of evaluation because they expect that option to offer more value than the less popular option would.

We find that in situations in which someone is focused on the quality aspects of products popularity decreases activity in the neural collective used to infer product quality and to examine the functional benefits of the option. Such a finding suggests an automatic link between popularity and quality. Parker and Lehmann (2011) noted that the link between popularity and quality seems so natural and automatic that people do not need to think about it. The results of this study are consistent with this argument; if people evaluate products the way they would normally do (i.e., normal focus), then there is more activity in the quality collective compared to when quality actually matters. This finding is further corroborated by the behavioral results. There is no indication that people have difficulties with drawing inferences about quality for popular products. Moreover, these drawn inferences may explain their own preferences (i.e., purchase intention) and subsequent behavior. This would suggest a link between popularity and choice that is explained via inferences revolving around quality. As such, we would argue that a large portion of the initial subjective value of popularity might be equated with quality and functional benefits.

On the social route, we find that activity in the social approval collective positively interacts with activity in the reward system. The regions of the brain that are part of the social approval collective are heavily involved with thinking about the states and opinions of others, both positive and negative ones. The neural ROIs that were selected for this study to represent social value may be engaged when people assess both positive and negative feedback from others with respect to their choices (Cascio et al., 2015). We argue that the interaction between the social approval collective and the reward system and the subsequent positive influence of this interaction on individual preferences indicate that people use popularity to infer a positive social outcome (i.e., expectations of social approval) for their choice of a popular product. Thus, people who use popularity for normative concerns would use popularity not out of concern for disapproval but out of expectations of approval.

Inferring cognitive processes from activation in particular areas of the brain (i.e., reverse inference) should be done with caution (Plassmann et al., 2015; Smeets et al., 2019). To minimize reverse inference in the current study we took the following approach: in line with the current best practices for food-related fMRI (Smeets et al., 2019) we had *a priori* ROIs and these were based on independent data (i.e., coordinates of earlier studies, using meta-analyses where available). Further, we combined the ROIs into a single combined ROI mask per construct and we used stringent statistical thresholds. This practice allowed us to narrow our focus on specific areas of the brain, as well as within areas of the brain. Some portions of the brain are rather large and not all of the area may be implicated in a particular behavior. For instance, the anterior insula (AI) has been implicated in diverse conditions and behaviors such as feeling satiated, perceiving time and estimating potential social approval (Craig, 2009; Baek et al., 2017). In the current study some areas of the AI were allocated to the ROI mask of the reward system whereas other areas were allocated to the ROI mask of the social approval collective. In doing so, we were able to discern the processes of how people use popularity cues to inform their decisions. The neural interpretation of those processes that we report here, is done on the level of clusters of brain areas implicated with popularity, valuation and choice.

### Practical Implications

These results have important implications for practitioners. Popularity is often used as a cue to persuade people. Our results indicate that the value associated with popular products may differ. Given the strong influence of popularity on behavior, marketers should carefully consider the focus of people that evaluate popular products. The results we present here indicate that for fast moving consumer goods, an emphasis on the social value may be particularly effective in stimulating sales. People’s intentions to purchase products are positively affected when they use popularity to assess a product’s social value and consider the opinions and reactions of others. People’s purchase intentions appear negatively affected if they use popularity to assess the quality of the product. Given this, it would seem advisable to emphasize the social benefits popular products rather than the product’s quality. Yet, future research can examine whether that advice holds in practice.

### Limitations and Directions for Future Research

A possible limitation of the current study is the way participants were manipulated to hold a particular focus. It may be possible that the method used to manipulate participants led to heightened activity in particular regions of the brain allocated to task management. Subsequently, this neural activity may have interfered with the activity in the collectives we proposed. For example, the activity in the lingual gyrus could be a result of the manipulation used. Our whole brain analyses (see Supplementary Data File) showed this region to be active not only in the instructed focus conditions (social and quality) when popularity was high, but also in the normal focus condition when popularity was low.

Participants reread the instructions pertaining to the focus manipulations throughout the entire run of the study (from training to post-scan task). Hare et al. (2011) successfully showed that people can rapidly shift focus to evaluate products. The current study was modeled after that study and increased the repetition of focus instructions to warrant a clear understanding of the stimuli and ensure a clear focus. However, the study did not include a manipulation check to measure the effectiveness of the focus instructions. As a result, no tests could be conducted to examine if the effect of a particular focus instruction was stronger (i.e., larger in effect size) than the effect of other instructions.

The current study has produced several negative results. One of these is that activity in the quality focus conditions negatively affected purchase intention compared to the normal focus condition. An alternative explanation of this result may point to a possible role of popularity as a simple heuristic that allows people to quickly draw inferences about a product without having to cognitively deliberate about the choice. The focus on quality of popular products may have led people to infer that popularity may not equal quality. Popular products cater to many heterogeneous tastes that do not necessarily reflect high quality. The current experimental setup does not allow for a proper account of those effects. Future research could shed further light on the role of popularity as a heuristic by specifically examining whether the effect is the result of deliberate reasoning or automatic decision making.

The stimuli we selected for the experiment are actual products, but they were not available in the local market (at least not at the time of data collection). The evaluations and manipulations were therefore hypothetical in nature. It would be interesting to examine whether the results obtained in the current study could be linked to actual decision making (i.e., consumer choices). For example, Berns and Moore (2012) found that activity in the reward system when listening to particular songs could be linked to the songs’ success in the hit charts. It would also be interesting for future research to examine whether the neural routes proposed here could also account for actual shopping behavior in supermarkets. Such research could aid in the development of new product campaigns.

## Data Availability Statement

The raw data supporting the conclusions of this article will be made available by the authors, without undue reservation.

## Ethics Statement

The studies involving human participants were reviewed and approved by Medical Ethical Committee of Wageningen University and registered in the Dutch Trial Registry (NTR5899). The participants provided their written informed consent to participate in this study.

## Author Contributions

RG, NL, and HT designed the study. RG supervised the data collection for all the pretests. RG and IT collected the data for the main study. RG analyzed the data with supervision and input from NL and HT. RG, NL, and HT discussed and interpreted the data throughout the entire research agenda. All authors contributed to the article and approved the submitted version.

## Conflict of Interest

IT is currently employed by company Innova Market Insights. The remaining authors declare that the research was conducted in the absence of any commercial or financial relationships that could be construed as a potential conflict of interest.

## Publisher’s Note

All claims expressed in this article are solely those of the authors and do not necessarily represent those of their affiliated organizations, or those of the publisher, the editors and the reviewers. Any product that may be evaluated in this article, or claim that may be made by its manufacturer, is not guaranteed or endorsed by the publisher.
