# Supplementary material for: The Subjective Value of Product Popularity: A Neural Account of How Product Popularity Influences Choice Using a Social and a Quality Focus
Source: Front Psychol. 2022 Jan 19;12:738095. doi: 10.3389/fpsyg.2021.738095 (PMC8809406; doi:10.3389/fpsyg.2021.738095)
Supplement: Supplementary file 1 [file Data_Sheet_1.docx]

Supplementary Material

**“The subjective value of popularity: A neural account of how popularity influences behavior using a social and quality focus”**

This document contains a report of the materials that supplement the manuscript of The subjective value of popularity: A neural account of how popularity influences behavior using a social and quality focus. First, there is a description of the different pretests we conducted in setting up the fMRI experiment. Next, there follows a detailed description of the technical details of the scanning equipment used for data acquisition and details concerning the data processing, and data extraction. Finally, we report the results of several whole brain analyses.

**Overview pretests**

**General Note**

At the beginning of all pretests, participants were welcomed and invited to read a consent form that described the content of the study and gave instructions about it. By proceeding, the participants indicated that they had read and understood the form and agreed to participate. In all studies, participants were given the contact details of the principal investigator (this paper’s corresponding author) in case they had additional questions. Participants were free to drop out at any time but none chose to do so. Finally, participants received compensation in the form of a snack. Participants were asked to sign for receipt of the compensation. All materials were presented in the participants’ native language and are translated to English in this Appendix.

**Pretest: Selection Target Group Description**

In order to create the right description for a target group with which the participants could identify, we asked a group of female participants (*n* = 113) to evaluate one of three different vignettes that described a group of consumers. Vignette 1 contained a simple description of characteristics. Vignette 2 contained narrative of a group of consumers, and the same for vignette 3.

*Vignette 1.* These are characteristics of a subject pool for a large study spanning different local universities.

- Female
- Native
- 18–35 years of age
- Student
- BMI: 18.5–25 kg/m2
- Right-handed
- Cooking for themselves (and others)
- Doing their own groceries (and for others)
- Regular users of supermarket products
- A varied dietary pattern
- Critical and selective consumer

*Vignette 2.* This research is part of a larger study that is conducted at several local universities. In this study we are interested in how consumers evaluate new products. The participants in this study are local female students. These students do their own groceries. Their grocery list is often diverse and includes products such as dairy drinks, breakfast cereals, vegetables, fruits, salty snacks, sweet snacks, and other types of snacks. Our participants are generally critical consumers, not only towards themselves, but also to others. They value variety and therefore buy a wide range of different types of products in the supermarket. Our participants clearly know what they want.

*Vignette 3.* This research is part of a larger study that is conducted by this university. In this study we are interested in how consumers evaluate new, unknown products. Participants in this study are local female students. These students do their own groceries. They cook for themselves and also for others, such as roommates or friends. Their grocery list is often diverse and includes products such as vegetables and fruit, as well as cottage cheese and granola bars. Our participants clearly know what they want. Not just for themselves but also for others. We often see this reflected in their preferences for particular types of products. The participants in general often agree about what they do and do not prefer.

*Measures.* First, participants evaluated the vignettes and rated them on belongingness and association (Escalas and Bettman 2005), and potential of influence on consumption-related preferences (adapted from Viswanathan, Childers, and Moore 2000). All items were phrased in the form of statements to which participants were asked to express their agreement (7-point scales, 1: completely disagree; 7: completely agree). Next, participants were asked to indicate which characteristics of the group they liked the most and which they liked the least.

Belongingness (*α_V1_* = .94 ; *α_V2_* = .89 ; *α_V3_* = .94)

- I consider myself to be this type of person.
- I belong to this group.
- I fit in with this group of people.

Association (*α_V1_* = .88 ; *α_V2_* = .92 ; *α_V3_* = .89)

- Association with this group would positively reflect on someone.
- I would like to be identified with this group and what they represent.
- I would like to be linked to this group and what they stand for.

Potential of influence on consumption-related preferences (*α_V1_* = .84 ; *α_V2_* = .91 ; *α_V3_* = .80)

- The preferences within this group influence my choice for a type of store.
- The preferences within this group influence my choice for products and brands.
- The preferences within this group influence my choice for different styles of products.

*Results.* The results of Oneway ANOVAs show that belongingness differed per vignette (F(2, 112) = 3.07, *p* = .05). Participants indicated that they felt the most belongingness for the first vignette (*M* = 5.82, *SD* = 1.22). This was significantly higher than their belongingness scores for vignette 3 (*M* = 5.20, *SD* = 1.17, *p* = .016) but not higher than their belongingness scores for vignette 2 (*M* = 5.43, *SD* = .91, *p* = .128). Participants’ association with the group in the vignette also differed per vignette (*F*(2, 112) = 5.36, *p* = .014). Participants associated most with the group from vignette 1 (*M* = 5.26, *SD* = 1.16). Association with the group described in this vignette was significantly higher than with the group in vignette 2 (*M* = 4.53. *SD* = 1.12, *p* = .005) and higher than with the group in vignette 3 (*M* = 4.74, *SD* = 1.03, *p* = .040). Participants did not feel that the groups would differently influence their consumption-related preferences (*F*(2, 112) = .62, *p* = .541).

The results of the open-ended questions show that participants found the independence (in cooking and doing groceries) of the people in vignette 1 most favorable. They also liked that the group had a varied dietary pattern and was critical/selective. These latter favorable remarks were also applied to the group in vignette 2. The participants did not like the statements regarding being critical towards others. This also applied to vignette 3: participants favored the independence and the varied dietary pattern but disliked the knowing not only for themselves but also for others. Participants did favor the ideas of being caring and social towards others.

Based upon these results we took the characteristics from vignette 1 and added the favored characteristics from vignettes 2 and 3 regarding being caring and social. The final target group was presented as follows:

*Target group description as presented to participants.*

“This research is part of a larger study that is conducted in several local universities. In this study we are interested in how consumers evaluate new products. The aim of this study is to gain more insights into the dietary patterns of consumers, which may include choices for new products. In previous experiments we asked participants to evaluate new products. All participants are female students. You can recognize them by the following characteristics:”

| Demographical | Lifestyle |
| --- | --- |
| - Female   - Native    - Age 18–35   - Student   -BMI: 20–25 kg/m2 | - Regular user supermarket products    - Cooking for themselves (and others)    - Doing their own groceries    - Varied dietary pattern   - Critical and selective (clear opinion)    - Caring and social |

**Pretest: Selection Popularity Cue**

In order to select an appropriate symbol to convey popularity, we asked a group of female participants (*n* = 50) to evaluate two types of popularity cues: a shopping basket with a percentage representing popularity or a shopping cart with a percentage representing popularity. Participants were told that they would participate in a study that involved evaluating salty snacks that were previously evaluated by other participants. The salty snacks in this study were labeled with a popularity score based on the previous study. Popularity was said to be expressed by a percentage, which is an effective method to convey popularity (Goldstein, Cialdini, and Griskevicius 2008). The products in the current survey were either low in popularity (25%–35%) or high in popularity (65%–75%). Participants were told that the percentage was displayed with either a shopping basket (condition 1) or a shopping cart (condition 2). Participants were randomly allocated to one of these two conditions. The evaluation was a cover story; we were only interested in participants’ understanding of the popularity cue.

In each of the conditions, participants evaluated four different salty snacks. Two of the products were low in popularity (25% and 35%) and two were high in popularity (65% and75 %). As part of the cover, participants evaluated each of the snacks on purchase intention (“I would like to buy this product”), quality (“This product is of a high quality”), and social identity (“This product is good for signaling a social identity”). Next, participants were asked what the symbol conveyed (open-ended question). Finally, participants were asked whether the symbol was a clear symbol for conveying popularity (“This is a clear symbol to convey popularity”), whether it was credible (“The symbol is a credible symbol to convey popularity”), and whether the symbol helped during the survey (“The symbol helped me to respond to the statements”). Participants were asked to express their agreement to all statements (9-point scales, 1: completely disagree; 7: completely agree).

*Results.* The participants in the condition with the shopping basket all correctly remembered (correct = 1, incorrect = 2) that the shopping basket conveyed popularity (100%). In the condition with the shopping cart, the number of participants that correctly remembered that the symbol conveyed popularity was lower (75%). This difference was significant (χ²(1) = 6.82, *p* = .009). Participants did not find the shopping cart clearer than the shopping basket (*F*(1, 49) = .01, *p* = .949), nor did they evaluate differently its credibility (*F*(1, 49) = .02, *p* = .890), or whether the symbol helped them (*F*(1, 49) = .54, *p* = .464). See table 1 for detailed results.

| **Table 1.** Mean Scores and Standard Deviation | | |
| --- | --- | --- |
|  | *Shopping basket* | *Shopping cart* |
| Clarity | 5.64 (1.89) | 5.60 (2.48) |
| Credibility | 4.76 (1.88) | 4.84 (2.19) |
| Helpful | 3.24 (1.67) | 3.64 (2.14) |
| Remembered correctly | 100% | 76% |

**Full instructions and manipulation**

Participants first received information about the setup of the study and were explained that they were to evaluate products with different types of focus. Similar to the procedure used by Van Meer and colleagues (2017), colors were used to ease learning about the different focus conditions and to aid during the task. The combinations between colors and focus conditions were counter-balanced across the experiment.

**Information**

In previous experiments conducted by this research project, female students evaluated a large number of new and unknown products. Their assignment was to judge the products on how suitable they were for different situations. Some products were evaluated *without specific instructions*. Other products were evaluated in terms of their suitability for situations in which *product quality* is very important or in which you *want to impress*. *Normal* focus is indicated with the color *blue*. A *quality focus* is indicated with *red*, and *social focus* with *green*.

As an example, you could think of preparations for a dinner party or a regular party. When you cook for yourself, you pay attention to other things than when you are expecting guests. In the latter kind of situations, it could be very important to serve *high quality* products or products that will *impress your friends* and help you get their *approval*. So, you can choose products with a *normal focus*, a *quality focus*, or a *social focus*. When you evaluate products with a *normal* focus, you evaluate them as you would *normally* do. When you evaluate products with a *quality focus,* you pay attention to the *quality* of the products and how *good* they are. When you evaluate products with a *social focus,* you think of the *social approval of your friends* and if you can make a *good impression* with what you buy.

Previous participants evaluated the products from the current study with each type of focus. Some products were evaluated very well and received a high score on **purchase intention**. A large portion of the respondents indicated that they **really wanted to buy** those products. The **purchase intention** of the participants is expressed with a percentage. The percentage indicates how much people wanted that product. The higher the percentage, the **higher the purchase intention**. Below you will find some examples of how purchase intention is displayed.

| Low purchase intention | | High purchase intention | |
| --- | --- | --- | --- |
| 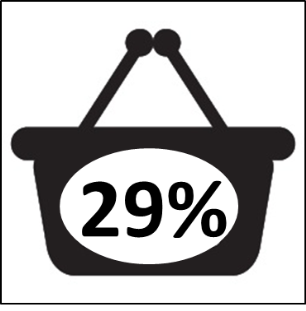 | 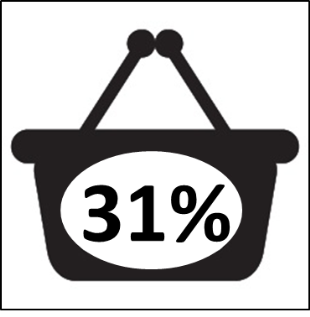 | 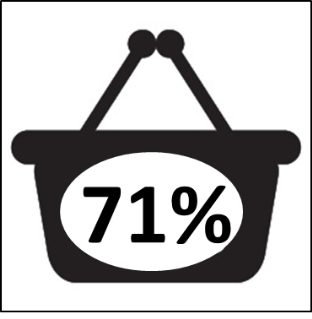 | 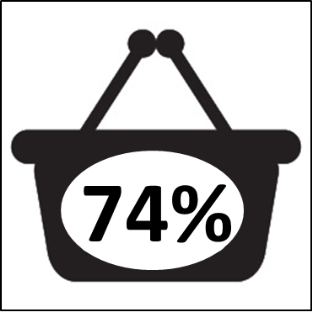 |

**Instructions for the task**

Please evaluate the products in this task with a specific focus. The type of focus with which you need to evaluate the products is indicated with a short instruction, at the end of which you will see the color of the focus condition. *Normal* focus is indicated with the color *blue*, a *quality focus* is indicated with *red*, and *social focus* with *green*.

The focus with which you will evaluate the products will change throughout the task. Evaluate each product with the focus of the condition. Next, please indicate your purchase intention for the product.

For particular products, you need to pay attention to *social* factors, such as the *social approval* from your *social context.* In the experiment, this focus is introduced with the instruction *social focus*.

For particular products, you need to pay attention to the relevance of *quality* or *taste*. These products are evaluated in terms of *quality.* In the experiment, this focus is introduced with the instruction *quality focus*.

Other products you can evaluate like you would *normally* do. In the experiment, this focus is introduced with the instruction *normal focus*.

**MRI Data collection and processing**

**MRI Data Acquisition**

Functional data were collected in two consecutive MRI sessions. There was a small pause between sessions to give participants a moment of rest. The length of the sessions varied slightly and contained on average 354 volumes (758 seconds). The functional images were acquired using a T2-weighted gradient echo-planar imaging sequence (TR = 2140ms, TE = 25ms, 90° flip order, FOV = 192*192mm, descending order, 43 axial slices, voxel size = 3*3*3mm) on a 3T Siemens Magnetom Verio (Siemens, Erlangen, Germany). We collected for each participant an additional anatomical scan (T1-weighted) (MPRAGE, TR = 2300ms, TE = 2.98ms, 9° flip angle, FOV = 256*256, 192 sagittal slices, voxel size = 1*1*1mm) after the collection of the functional scans.

**MRI Data Processing**

To prepare the data for analysis, the brain images were preprocessed with SPM12 (Wellcome Department of Imaging Neuroscience, London, UK). Functional images of every participant were slice-time corrected to correct for time differences between respondents. Next, the images were realigned to the first volume of the first run to account for movement in the scanner. To allow for comparison between functional scans and the participants’ anatomy within and beyond the sample, the functional and anatomical images were coregistered and globally normalized to the Montreal Neurological Institute space (MNI-space). To remove small anatomical differences, the images were spatially smoothed with a Gaussian kernel of 6mm full-width at half maximum. Next, to link timing of the events with the participants’ biological reaction, a statistical parametric map was generated for every subject by fitting a boxcar function to each time series, convolved with the canonical hemodynamic response function. Data was high-pass filtered (cut-off: 128s) to remove low-frequency noise that could lead to false positives.

**Overview Regions of interest**

This section presents a full overview of the peak coordinates that were taken from previous studies (see table 2 for full list).

| **Table 2.** Overview coordinates ROI Masks | | | |
| --- | --- | --- | --- |
| **Reward** |  | | |
| *Region* | *X* | *Y* | *Z* |
| Striatum (L) | -12 | 12 | -6 |
| Striatum (R) | 14 | 12 | -10 |
| vmPFC | 2 | 46 | -8 |
| PCC | 2 | -36 | 32 |
| Anterior insula (L) | -32 | 26 | 0 |
| Anterior insula (R) | 32 | 20 | -6 |
| Brainstem | 0 | -20 | -10 |
| ACC | -2 | 24 | 26 |
| **Quality** |  | | |
| *Region* | *X* | *Y* | *Z* |
| Medial Orbitofrontal cortex | -1 | 27 | -18 |
| Inferior occipital gyrus (L) | -45 | -70 | -13 |
| Middle temporal gyrus (R) | 45 | -58 | 6 |
| Middle occipital gyrus (L) | -12 | -94 | -1 |
| Fusiform gyrus (R) | 42 | -46 | -20 |
| Cingulate gyrus (R) | 18 | 11 | 29 |
| Parahippocampal gyrus (L) | -30 | -1 | -24 |
| Cuneus (R) | 24 | -82 | 16 |
| **Approval** |  | | |
| *Region* | *X* | *Y* | *Z* |
| mmPFC (bilateral) | -9 | 59 | 4 |
| dmPFC | -18 | 38 | 43 |
| TPJ (L) | -51 | -64 | 34 |
| Precuneus (L) | -9 | -55 | 19 |
| Inferior temporal gyrus | 66 | -10 | -14 |
| Middle temporal gyrus | -60 | -10 | -17 |
| Insula (L) | -33 | 45 | 36 |
| Cingulate (L) | -9 | 18 | 42 |

Based upon these studies’ coordinates the regions of interest for the current study were constructed. The coordinates for the reward value mask were taken from the analysis on positively valenced outcomes by Bartra and colleagues (2013). In that analysis, the authors control for activity at the time of negatively valenced outcomes. The coordinates for the quality mask were derived from Hare and colleagues (2008) and Couwenberg and colleagues (2017). In the latter study, the authors list coordinates of brain regions that are active when people evaluate functional benefits while controlling for experiential benefits. Finally, the coordinates for the approval regions were derived from Baek and colleagues (2017) who investigated social valuation during decision making (i.e. selection phase). The approval coordinates were complemented with coordinates for the anterior insula (AI) and anterior cingulate cortex (ACC) from Berns and colleagues (2010). In their study they note activation during listening to popular songs while controlling for listening to unpopular songs.

**Whole brain analyses; model with purchase intention as parametric modulator**

This section contains an overview of the significant clusters found with whole brain analyses per condition. All analyses were conducted using a threshold of *p* < .005, with *k* > 20.

| **Table 3. Main effect of popularity** | | | | | | | | |
| --- | --- | --- | --- | --- | --- | --- | --- | --- |
| **Popularity low** |  |  | MNI coordinates | | |  |  |  |
|  | ***L*** |  | ***x*** | ***y*** | ***z*** | ***k*** | ***Z*** | ***p*** |
| Dorsal anterior cingulate | R | | 6 | 38 | 35 | 45 | 4.14 | .000 |
| **Popularity high** | | | | | | | | |
| Inf. frontal gyrus |  | | -27 | 2 | 35 | 20 | 3.83 | .000 |
| Calcarine* |  | | 6 | -88 | 5 | 114 | 3.73 | .000 |
| *Occipital gyrus* |  | | 9 | -88 | 26 |  | 3.56 | .000 |
|  |  | | 18 | -85 | 32 |  | 2.85 | .000 |
| Lingual gyrus | L | | -15 | -64 | -13 | 20 | 3.63 | .000 |
| **p_FWE_ <* .05 at cluster-level, † *p_FWE_* < .10 at cluster-level | | | | | | | | |

For the main effect of popularity we find activity in the calcarine, known for visual processing. Also, we find some activity in the inferior frontal gyrus and the lingual gyrus. These clusters are not that large. For low popularity we find activity in the dorsal anterior cingulate cortex, known for various cognitive functions, including emotional expression, attention allocation, and mood regulation.

| **Table 4. Main effect of focus** | | | | | | | | |
| --- | --- | --- | --- | --- | --- | --- | --- | --- |
| **Quality vs normal** |  |  | MNI coordinates | | |  |  |  |
|  | ***L*** |  | ***x*** | ***y*** | ***z*** | ***k*** | ***Z*** | ***p*** |
| Intraparietal sulcus | L | | -39 | -55 | 56 | 27 | 3.47 | .000 |
| Superior temporal sulcus | L | | -60 | -46 | 2 | 47 | 3.41 | .000 |
| *Mid. temporal gyrus* | L | | -57 | -40 | -4 |  | 3.19 | .001 |
| **Social vs normal** | | | | | | | | |
| Supramarginal gyrus | L | | -54 | -46 | 29 | 31 | 3.50 | .000 |
| *Superior temporal sulcus* | L | | -57 | -49 | 20 |  | 3.16 | .001 |
|  | L | | -45 | -37 | 32 |  | 2.97 | .001 |
| Mid. frontal gyrus | L | | -42 | 26 | 44 | 30 | 3.36 | .000 |
|  | L | | -48 | 14 | 47 |  | 3.07 | .001 |
| Mid. frontal gyrus | R | | 39 | 20 | 35 | 20 | 3.19 | .001 |
| **p_FWE_ <* .05 at cluster-level, † *p_FWE_* < .10 at cluster-level | | | | | | | | |

In the quality condition, while controlling for the normal condition, we find a relatively large cluster in the superior temporal sulcus, known for visualizing social information. We also find this for the social context, while controlling for the normal focus condition.

| **Table 5. Interaction effects; normal focus & low popular vs. quality focos & high popularu** | | | | | | | | |
| --- | --- | --- | --- | --- | --- | --- | --- | --- |
| **‘Normal & low’ < ‘Quality & high’** |  |  | MNI coordinates | | |  |  |  |
|  | ***L*** |  | ***x*** | ***y*** | ***z*** | ***k*** | ***Z*** | ***p*** |
|  | No suprathreshold clusters | | | | | | | |
| **‘Normal & low’ > ‘Quality & high’** | | | | | | | | |
| Sup. Frontal gyrus | R | 9 | | 35 | 38 | 24 | 3.43 | .000 |
|  |  | 18 | | 41 | 38 |  | 3.02 | .001 |
| **p_FWE_ <* .05 at cluster-level | | | | | | | | |

In the contrast between popularity low under a normal focus and popularity high under a quality focus we only find a small cluster in the superior frontal gyrus for low popularity under normal focus.

| **Table 6. Interaction effects; normal focus & low popular vs. social focus & high popular** | | | | | | | | |
| --- | --- | --- | --- | --- | --- | --- | --- | --- |
| **‘Normal & low’ < ‘Social & high’** |  |  | MNI coordinates | | |  |  |  |
|  | ***L*** |  | ***x*** | ***y*** | ***z*** | ***k*** | ***Z*** | ***p*** |
| Superior temporal sulcus | L |  | -57 | -49 | 17 | 48 | 3.95 | .000 |
| Intraparietal sulcus | L |  | -36 | -61 | 56 | 66 | 3.57 | .000 |
| *Inf. parietal gyrus* | L |  | -45 | -52 | 53 |  | 3.19 | .001 |
| *Sup. parietal gyrus* | L |  | -33 | -55 | 62 |  | 3.07 | .001 |
| **‘Normal & low’ > ‘Social & high’** | | | | | | | | |
|  | No suprathreshold clusters | | | | | | | |
| **p_FWE_ <* .05 at cluster-level | | | | | | | | |

In the condition of high popularity and social focus we find clusters in the superior temporal sulcus and the intraparietal sulcus. These are both regions known to be involved in processing the behavior and opinions of others.

| **Table 7. Interaction effects; normal focus & high popular vs. quality focus & low popular** | | | | | | | | |
| --- | --- | --- | --- | --- | --- | --- | --- | --- |
| **‘Normal & high’ < ‘Quality & low’** |  |  | MNI coordinates | | |  |  |  |
|  | ***L*** |  | ***x*** | ***y*** | ***z*** | ***k*** | ***Z*** | ***p*** |
| Superior frontal sulcus | L |  | -33 | 26 | 29 | 43 | 4.34 | .000 |
| Superior frontal gyrus* | L |  | -12 | 23 | 62 | 346 | 4.19 | .000 |
|  | R |  | 3 | 47 | 53 |  | 3.82 | .000 |
|  | L |  | -9 | 50 | 47 |  | 3.80 | .000 |
| Superior frontal sulcus | R |  | 24 | 50 | 44 | 31 | 4.08 | .000 |
|  | R |  | 33 | 41 | 44 |  | 3.19 | .001 |
| Mid. frontal gyrus | L |  | -36 | 17 | 56 | 67 | 3.95 | .000 |
| *Superior frontal sulcus* | L |  | -33 | 8 | 59 |  | 3.47 | .000 |
|  | L |  | -27 | 14 | 62 |  | 3.42 | .000 |
| Temporal inf. gyrus | L |  | -63 | -52 | -4 | 31 | 3.91 | .000 |
|  | L |  | -60 | -46 | 2 |  | 3.43 | .000 |
| Superior frontal sulcus | R |  | 18 | 8 | 56 | 26 | 3.78 | .000 |
|  | R |  | 18 | -1 | 47 |  | 2.93 | .002 |
|  | R |  | 24 | 17 | 56 |  | 2.79 | .003 |
| Mid temporal pole | R |  | 57 | -1 | -31 | 23 | 3.68 | .000 |
| Inf. frontal gyrus | R |  | 54 | 44 | -7 | 35 | 3.57 | .000 |
|  |  |  | 54 | 35 | -10 |  | 3.45 | .000 |
|  |  |  | 45 | 53 | -13 |  | 3.35 | .000 |
| Medial orbitofrontal gyrus | L |  | -45 | 44 | -16 | 23 | 3.55 | .000 |
| **‘Normal & high’ > ‘Quality & low’** | | | | | | | | |
| No suprathreshold clusters | | | | | | | | |
| **p_FWE_ <* .05 at cluster-level | | | | | | | | |

We find a lot of activity in the popular high under normal focus condition when we control for activity in the low popular under quality focus condition. The majority of activity appears to be in the frontal regions of the brain consisting of the superior frontal sulcus and superior frontal gyrus. This large region is known for various functions such as the working memory, self-awareness as well as motor activity.

| **Table 8. Interaction effects; normal focus & high poplar vs. social focus & low popular** | | | | | | | | |
| --- | --- | --- | --- | --- | --- | --- | --- | --- |
| **‘Normal & high’ < ‘Social & low’** |  |  | MNI coordinates | | |  |  |  |
|  | ***L*** |  | ***x*** | ***y*** | ***z*** | ***k*** | ***Z*** | ***p*** |
| Cingulate | R | | 6 | 23 | 29 | 65 | 4.10 | .000 |
| *Frontal sup. medial gyrus* | R | | 3 | 38 | 38 |  | 3.75 | .000 |
| Anterior insula | R | | 30 | 23 | -1 | 38 | 4.48 | .000 |
| *Rolandic operculum* | R | | 36 | 11 | 11 |  | 3.08 | .001 |
| *Anterior insula* | R | | 30 | 14 | 5 |  | 3.02 | .001 |
| **‘Normal & high’ > ‘Social & low’** | | | | | | | | |
| Inf. frontal sulcus | L | | -24 | -1 | 35 | 56 | 4.74 | .000 |
|  | L | | -36 | -7 | 35 |  | 4.23 | .000 |
|  | L | | -48 | -4 | 38 |  | 3.28 | .001 |
| Olfactory sulcus | R | | 6 | 41 | -22 | 52 | 3.83 | .000 |
| *Subcallosal gyrus* | L | | -9 | 29 | -19 |  | 3.80 | .000 |
| Occipital lateral gyrus | R | | 21 | -91 | 11 | 59 | 3.53 | .000 |
|  | R | | 30 | -85 | 8 |  | 3.42 | .000 |
| Occipital lateral gyrus | R | | 36 | -76 | -22 | 26 | 3.30 | .000 |
|  | R | | 39 | -70 | -13 |  | 3.23 | .001 |
| Occipital lateral gyrus | L | | -21 | -94 | 14 | 26 | 3.30 | .000 |
| *Cuneus* | L | | -9 | -94 | 14 |  | 2.95 | .002 |
| **p_FWE_ <* .05 at cluster-level, † *p_FWE_* < .10 at cluster-level | | | | | | | | |

In the social focus low popularity condition, we find activity in the cingulate cortex and the anterior insula. These regions are known to be involved in processing negative social emotions (e.g., fear of disapproval). In the opposite contrast, high popular and normal focus we find activity in areas known for processing language and speech, connecting senses, and object recognition.

| **Table 9.** Results whole brain analysis focus condition: normal focus | | | | | | | | |
| --- | --- | --- | --- | --- | --- | --- | --- | --- |
| **Popularity low** |  |  | MNI coordinates | | |  |  |  |
|  | ***L*** |  | ***x*** | ***y*** | ***z*** | ***k*** | ***Z*** | ***p*** |
| Lingual gyrus^*^ | R | | 6 | -73 | -4 | 263 | 4.36 | < .001 |
|  | R | | 15 | -70 | -10 |  | 4.26 | < .001 |
| *Calcarine* | R | | 18 | -91 | 5 |  | 3.99 | < .001 |
| Superior frontal gyrus^†^ | L | | -21 | 35 | 56 | 92 | 3.95 | < .001 |
|  | L | | -21 | 17 | 59 |  | 3.65 | < .01 |
|  | L | | -15 | 26 | 59 |  | 3.22 | < .01 |
| Orbital / olfactory sulcus | L | | -24 | 29 | -19 | 22 | 3.54 | < .001 |
|  | L | | -3 | 26 | -22 |  | 3.07 | < .01 |
|  | L | | -15 | 38 | -19 |  | 2.88 | < .01 |
| Inferior frontal sulcus | L | | -42 | 41 | 8 | 20 | 3.50 | < .001 |
| Posterior cingulate cortex | L | | -6 | -40 | 29 | 30 | 3.34 | < .001 |
|  | L | | -15 | -49 | 35 |  | 2.98 | < .01 |
|  | L | | -9 | -32 | 29 |  | 2.89 | < .01 |
| **Popularity high** | | | | | | | | |
| Calcarine^*^ | R | | 9 | -73 | 2 | 94 | 3.76 | < .001 |
|  | R | | 3 | -88 | 2 |  | 3.33 | < .001 |
| *Fusiform* | R | | 18 | -76 | -10 |  | 3.32 | < .001 |
| **p_FWE_ <* .05 at cluster-level, † *p_FWE_* < .10 at cluster-level | | | | | | | | |

In the low popularity condition, there was high activation in the lingual gyrus, which is known to be involved in visual attention and word processing (e.g., memorizing, memory retrieval). There was also a lot of activation in the superior frontal gyrus, which is known to be involved with self-awareness in coordination with the sensory system.

| **Table 10.** Results whole brain analysis focus condition: quality focus | | | | | | | | |
| --- | --- | --- | --- | --- | --- | --- | --- | --- |
| **Popularity low** |  |  | MNI coordinates | | |  |  |  |
|  | ***L*** |  | ***x*** | ***y*** | ***z*** | ***k*** | ***Z*** | ***p*** |
| Superior frontal sulcus | L | | -21 | 44 | 44 | 69 | 3.42 | < .001 |
|  | L | | -12 | 47 | 38 |  | 3.22 | < .01 |
| Inferior frontal gyrus | L | | -33 | 23 | 32 | 40 | 3.12 | < .01 |
|  | L | | -39 | 5 | 29 |  | 2.89 | < .01 |
|  | L | | -42 | 14 | 32 |  | 2.85 | < .01 |
| **Popularity high** | | | | | | | | |
| Lingual gyrus^*^ | R | | 9 | -76 | -4 | 217 | 4.77 | < .001 |
|  | R | | 12 | -67 | 2 |  | 4.08 | < .001 |
| *Calcarine* | R | | 6 | -88 | 5 |  | 3.75 | < .001 |
| Superior frontal sulcus | L | | -15 | 26 | 56 | 28 | 3.21 | < .01 |
|  | L | | -24 | 26 | 56 |  | 2.96 | < .01 |
| **p_FWE_ <* .05 at cluster-level, † *p_FWE_* < .10 at cluster-level | | | | | | | | |

In the quality focus condition, there was a lot of activity in the lingual gyrus for products that score high in popularity but not for those that score low in popularity. This finding is in contrast to the results obtained for the normal focus.

| **Table 11.** Results whole brain analysis focus condition: social focus | | | | | | | | |
| --- | --- | --- | --- | --- | --- | --- | --- | --- |
| **Popularity low** |  |  | MNI coordinates | | |  |  |  |
|  | ***L*** |  | ***x*** | ***y*** | ***z*** | ***k*** | ***Z*** | ***p*** |
| Cingulate cortex | R | | 6 | 23 | 29 | 25 | 3.66 | < .001 |
|  | L | | -9 | 26 | 29 |  | 3.04 | < .01 |
| **Popularity high** | | | | | | | | |
| Lingual gyrus^*^ | R | | 9 | -73 | -1 | 685 | 4.29 | < .001 |
|  | R | | 18 | -70 | 14 |  | 4.06 | < .001 |
| *Calcarine* | R | | 3 | -94 | 8 |  | 3.86 | < .001 |
| Inf. parietal gyrus^†^ | L | | -36 | -64 | 53 | 97 | 4.00 | < .001 |
| *Angular gyrus* | L | | -30 | -73 | 44 |  | 3.68 | < .001 |
| *Sup. parietal gyrus* | L | | -18 | -67 | 50 |  | 2.95 | < .01 |
| Sup. Temporal sulcus | L | | -57 | -49 | 20 | 36 | 3.65 | < .001 |
| Mid. Temporal gyrus | L | | -57 | -40 | -13 | 20 | 3.56 | < .001 |
| Mid. Frontal gyrus | L | | -36 | 11 | 38 | 41 | 3.42 | < .001 |
|  | L | | -45 | 20 | 47 |  | 3.27 | < .01 |
| Occipital lateral gyrus | L | | -24 | -79 | -1 | 28 |  |  |
|  | L | | -39 | -85 | -4 |  |  |  |
| **p_FWE_ <* .05 at cluster-level, † *p_FWE_* < .10 at cluster-level | | | | | | | | |

In the social focus condition, there was again a lot of activity in the lingual gyrus when the participants evaluated products high in popularity.

**MRI Normal focus**

Parameters were extracted via Marsbar from contrasts in which activity in the normal focus condition was corrected for the activity in the quality focus and social focus conditions (model 1 without purchase intention as parametric modulator). Parameters were extracted from the following conditions: “normal focus and popularity high” versus ”social focus and popularity high;” “normal focus and popularity low” versus “social focus and popularity low;” “normal focus and popularity high” versus ”quality focus and popularity high;” “normal focus and popularity low” versus “quality focus and popularity low.”

*MRI Normal focus vs social focus*

First, we examined the neural correlates of popularity in the normal focus condition and examined the activation in the a priori defined regions of interest in the normal focus condition and compared this to activation in the social focus condition. In a similar fashion as reported in the paper, we conducted a multilevel mediation analysis on the scores of purchase intention that were collected in the scan-task. Popularity (*X_Pop_*) was entered as an independent variable, with the following mediators: parameters of activity in the social approval network (*ME_ROI_Soc_*), parameters of activity in the quality network (*ME_ROI_Qual_*), parameters of activity in the reward network (*ME_ROI_Rew_*), and interactions between the parameters of social approval and reward (*ME_ROI_Soc_* x *ME_ROI_Rew_*) and quality and reward (*ME_Qual_* x *ME_Rew_*). Different parts of this model were analyzed in a stepwise fashion to examine mediation before we ran the full model (see table 12 for a full overview of results).

**Table 12.** Parameters and test statistics normal focus controlled for social focus

| **Mediators only; Y = Intent** |  | | | | |
| --- | --- | --- | --- | --- | --- |
| *Fixed Parameters* | *β* | *SE* | *df* | *t* | *p* |
| intercept | -.323 | .099 | 32.604 | -3.260 | .003 |
| X_Pop_ | .327 | .041 | 1796.873 | 7.996 | .000 |
| ME_Qual_ROI_ | .263 | .138 | 117.433 | 1.909 | .059 |
| ME_Soc_ROI_ | .060 | .077 | 178.143 | .772 | .441 |
| ME_Rew_ROI_ | -.307 | .161 | 99.947 | -1.910 | .059 |
| *Covariance Parameters* | *β* | *SE* | *Wald Z* | | *p* |
| Error term – model | 2.805 | .094 | 29.740 | | .000 |
| Error term – intercept | .224 | .071 | 3.148 | | .002 |
| **Mediators only; Y = Reward ROI** | | | | | |
| *Fixed Parameters* | *β* | *SE* | *df* | *t* | *p* |
| intercept | -.063 | .080 | 30.063 | -.793 | .434 |
| X_Pop_ | -.017 | .004 | 1772.225 | -4.117 | .000 |
| ME_Qual_ROI_ | .466 | .016 | 1796.153 | 29.003 | .000 |
| ME_Soc_ROI_ | .326 | .007 | 1788.632 | 49.121 | .000 |
| *Covariance Parameters* | *β* | *SE* | *Wald Z* | | *p* |
| Error term – model | .028 | .001 | 29.749 | | .000 |
| Error term – intercept | .190 | .049 | 3.862 | | .000 |
| **Full model incl. interactions** |  | | | | |
| *Fixed Parameters* | *β* | *SE* | *df* | *t* | *p* |
| intercept | -.345 | .109 | 35.728 | -3.160 | .003 |
| X_Pop_ | .328 | .041 | 1785.937 | 7.994 | .000 |
| ME_Qual_ROI_ | .237 | .142 | 125.278 | 1.671 | .097 |
| ME_Soc_ROI_ | .076 | .080 | 155.728 | .949 | .344 |
| ME_Rew_ROI_ | -.214 | .177 | 130.861 | -1.209 | .229 |
| ME_Qual_ROI_ x ME_Rew_ROI_ | .301 | .163 | 378.228 | 1.847 | .066 |
| ME_Soc_ROI_ x ME_Rew_ROI_ | -.117 | .069 | 775.277 | -1.708 | .088 |
| *Covariance Parameters* | *β* | *SE* | *Wald Z* | | *p* |
| Error term – model | 2.806 | .094 | 29.710 | | .000 |
| Error term – intercept | .251 | .080 | 3.124 | | .002 |

An examination of the main effects on purchase intention shows that popularity significantly contributes to purchase intention (*βX_Pop_* = .327, *p* < .001). Activity in the quality network contributes positively as well, albeit marginally (*βME_ROI_Qual_* = .263, *p* = .059). Activity in the reward system also has a marginal negative contribution to purchase intention (*βME_ROI_Rew_* = -.307, *p* = .059). Activitiy in the social approval network did not affect purchase intention (*βME_ROI_Soc_* = .060, *p* = .441). Next, we analyzed the relation between activity in the regions of social approval and reward value and activity in the regions of quality and reward value. We find positive effects of both the social approval network (*βME_ROI_Soc_* = .326, *p* < .001) and the quality network (*βME_ROI_Qual_* = .466, *p* < .001). Popularity negatively affected activity in the reward system (*βX_Pop_* = -.017, *p* < .001).

For the final model that included all mediators and interactions, the results showed that activity in the social approval network did not contribute to purchase intention (*βME_ROI_Soc_* = .076, *p* = .344). Activitity in the social approval network did interact with activity in the reward system (*βME_ROI_Soc_* x *ME_ROI_Rew_* = -.117, *p* = .088) and had a marginal negative effect on purchase intention scores. Activity in the quality network contributed positively, albeit marginally (*βME_ROI_Qual_* = .237, *p* = .097). The activity in the quality network also interacted with activity in the reward system and positively contributed to purchase intention scores, albeit marginally (*βME_ROI_Qual_* x *ME_ROI_Rew_* = .301, *p* = .066). Activity in the reward system did not contribute to purchase intention (*βME_ROI_Rew_* = -.214, *p* = .229). The main effect of popularity remained significant (*βX_Pop_* = .328, *p* < .001).

Thus, in the normal focus condition, when controlling for activity in the social focus condition, the effect of product popularity on purchase intention is partially explained by activity in the quality network and by activation in that system that interacts with activity in the reward system. These parameters contribute positively to purchase intention scores. We also find a negative interaction between activity in the social approval network and the reward system. These results should be interpreted with caution because these predictors were only marginally significant.

*MRI Normal focus vs quality focus*

The neural correlates of popularity in the normal focus condition while controlling for activity in the quality focus condition were examined using a similar approach as describe above. Here, we examined the activation in our set of a priori defined regions of interest in the normal focus condition and compared this to activation in the quality focus condition (see table 13 for a full overview of results).

**Table 13.** Parameters and test statistics normal focus controlled for quality focus

| **Mediators only; Y = Intent** |  | | | | |
| --- | --- | --- | --- | --- | --- |
| *Fixed Parameters* | *β* | *SE* | *df* | *t* | *p* |
| intercept | -.438 | .125 | 30.700 | -3.517 | .001 |
| X_Pop_ | .305 | .041 | 1799.768 | 7.454 | .000 |
| ME_Qual_ROI_ | -.149 | .145 | 226.363 | -1.025 | .306 |
| ME_Soc_ROI_ | -.032 | .073 | 499.111 | -.443 | .658 |
| ME_Rew_ROI_ | .413 | .165 | 336.164 | 2.495 | .013 |
| *Covariance Parameters* | *β* | *SE* | *Wald Z* | | *p* |
| Error term – model | 2.776 | .093 | 29.695 | | .000 |
| Error term – intercept | .356 | .115 | 3.110 | | .002 |
| **Mediators only; Y = Reward ROI** | | | | | |
| *Fixed Parameters* | *β* | *SE* | *df* | *t* | *p* |
| intercept | .040 | .066 | 30.505 | .597 | .555 |
| X_Pop_ | -.015 | .005 | 1773.811 | -3.012 | .003 |
| ME_Qual_ROI_ | .334 | .020 | 1792.890 | 16.602 | .000 |
| ME_Soc_ROI_ | .324 | .007 | 1798.090 | 49.718 | .000 |
| *Covariance Parameters* | *β* | *SE* | *Wald Z* | | *p* |
| Error term – model | .042 | .001 | 29.748 | | .000 |
| Error term – intercept | .129 | .034 | 3.848 | | .000 |
| **Full model incl. interactions** |  | | | | |
| *Fixed Parameters* | *β* | *SE* | *df* | *t* | *p* |
| intercept | -.423 | .138 | 28.494 | -3.062 | .005 |
| X_Pop_ | .327 | .043 | 1776.804 | 7.619 | .000 |
| ME_Qual_ROI_ | -.110 | .162 | 245.404 | -.681 | .497 |
| ME_Soc_ROI_ | .006 | .077 | 676.097 | .075 | .940 |
| ME_Rew_ROI_ | .575 | .176 | 390.968 | 3.273 | .001 |
| ME_Qual_ROI_ x ME_Rew_ROI_ | .019 | .113 | 498.123 | .174 | .862 |
| ME_Soc_ROI_ x ME_Rew_ROI_ | -.096 | .047 | 682.431 | -2.034 | .042 |
| *Covariance Parameters* | *β* | *SE* | *Wald Z* | | *p* |
| Error term – model | 2.766 | .093 | 29.657 | | .000 |
| Error term – intercept | .458 | .148 | 3.092 | | .002 |

The results of the direct effects revealed that activity in the quality regions did not contribute to purchase intention scores (*ME_ROI_Qual_* = -.149, *p* = .306), nor did activity in the social approval network (*βME_ROI_Soc_* = -.032, *p* = .658). Activity in the reward system did positively contribute to purchase intention (*βME_ROI_Rew_* = .413, *p* = .013). Popularity also positively affected purchase intention (*βX_Pop_* = .305, *p* < .001). The results of the activity in the reward system showed that activity in the quality network evoked more activity in the reward system (*ME_ROI_Qual_* = .334, *p* < .001), and activity in the social approval network evoked more activity in the reward system (*βME_ROI_Soc_* = .324, *p* < .001). Albeit small, popularity negatively affected activity in the reward value system (*βX_Pop_* = -.015, *p* = .003). The full model, which included all effects and interactions, shows that activity in the reward system positively contributed to purchase interaction scores (*βME_ROI_Rew_* = .575, *p* = .001). Activity in the reward system also interacted with activity in the social approval network but negatively affected purchase intention (*βME_ROI_Soc_* x *ME_ROI_Rew_* = -.096, *p* = .042). The main effect of popularity remained significant (*βX_Pop_* = .327, *p* < .001). None of the other predictors reached significance (*p*s > .10). Thus, when controlling for activity in the quality focus, we find that the effect of popularity on purchase intention scores is partially explained by activity in the reward system.

**References for supplementary materials**

Baek, E. C., Scholz, C., O’Donnell, M. B., & Falk, E. B. (2017). The Value of Sharing Information: A Neural Account of Information Transmission. *Psychological Science*, *28*(7), 851–861. http://doi.org/10.1177/0956797617695073

Bartra, O., McGuire, J. T., & Kable, J. W. (2013). The valuation system: A coordinate-based meta-analysis of BOLD fMRI experiments examining neural correlates of subjective value. *NeuroImage*, *76*, 412–427. http://doi.org/10.1016/j.neuroimage.2013.02.063

Berns, G. S., Capra, C. M., Moore, S. E., & Noussair, C. (2010). Neural mechanisms of the influence of popularity on adolescent ratings of music. *NeuroImage*, *49*(3), 2687–2696. http://doi.org/10.1016/j.neuroimage.2009.10.070

Couwenberg, L. E., Boksem, M. A. S., Dietvorst, R. C., Worm, L., Verbeke, W. J. M. I., & Smidts, A. (2017). Neural responses to functional and experiential ad appeals : Explaining ad effectiveness. *International Journal of Research in Marketing*, *34*(2), 355–366. http://doi.org/10.1016/j.ijresmar.2016.10.005

Escalas, J. E., & Bettman, J. R. (2005). Self‐Construal, Reference Groups, and Brand Meaning. *Journal of Consumer Research*, *32*(3), 378–389. http://doi.org/10.1086/497549

Goldstein, N. J., Cialdini, R. B., & Griskevicius, V. (2008). A room with a viewpoint: Using social norms to motivate environmental conservation in hotels. *Journal of Consumer Research*, *35*(3), 472–482. http://doi.org/10.1086/586910

Hare, T. A., O’Doherty, J., Camerer, C. F., Schultz, W., & Rangel, A. (2008). Dissociating the Role of the Orbitofrontal Cortex and the Striatum in the Computation of Goal Values and Prediction Errors. *Journal of Neuroscience*, *28*(22), 5623–5630. http://doi.org/10.1523/JNEUROSCI.1309-08.2008

van Meer, F., van der Laan, L. N., Viergever, M. A., Adan, R. A. H., & Smeets, P. A. M. (2017). Considering healthiness promotes healthier choices but modulates medial prefrontal cortex differently in children compared with adults. *NeuroImage*, *159*(July), 325–333. http://doi.org/10.1016/j.neuroimage.2017.08.007

Viswanathan, M., Childers, T. L., & Moore, E. S. (2000). The Measurement of Intergenerational Communication and Influence on Consumption: Development, Validation, and Cross-Cultural Comparison of the IGEN Scale. *Journal of the Academy of Marketing Science*, *28*(3), 406–424. http://doi.org/10.1177/0092070300283008
